# KNEE ARTHROPLASTY REVISION WITH A CONSTRAINED IMPLANT USING HINGE AND ROTATING TIBIAL BASIS

**DOI:** 10.1590/1413-785220162401153984

**Published:** 2016

**Authors:** Fabio Jansen Angelini, Camilo Partezani Helito, Bruno Azevedo Veronesi, Tales Mollica Guimarães, José Ricardo Pécora, Marco Kawamura Demange

**Affiliations:** 1. Universidade de São Paulo, Faculdade de Medicina, Department of Orthopedics and Traumatology, Knee Surgery Group, São Paulo, SP, Brasil.

**Keywords:** Arthroplasty, replacement, knee, Prostheses and Implants

## Abstract

**Objective:**

To evaluate the results of total knee arthoplasty revisions performed in high complexity cases, with large bone defects or serious ligament deficiencies using a constrained implant hinge associated to a rotating tibial basis.

**Methods:**

We evaluated 11 patients in which we used the constrained implant hinge associated to rotating tibial basis, with minimum follow-up of two years. The indications for the procedure included instability, septic loosening, late postoperative infection without loosening and periprosthetic fracture. We evaluated the knee range of movement and functional outcomes by the Knee Society Score (KSS) e Knee Injury and Osteoarthritis Outcome Score (KOOS), besides the presence of complications.

**Results:**

All patients achieved 5^o^ to 85^o^ minimum range of motion at 1 year postoperatively and, in the present evaluation, KSS ranged from 67 to 95. Three patients had no complications until the last evaluation and two patients required implant revision.

**Conclusion:**

Despite the complications rate observed, the functional result were acceptable for most patients, and it proved being a viable alternative, especially for patients with low functional demand. ***Level of Evidence IV, Case Series.***

## INTRODUCTION

Due to the aging of the population and consequently the increase of patients osteoarthritis, indication of total knee arthroplasty (TKA) is increasingly frequent.^1^ This procedure shows excellent postoperative results regarding survival of the implant, with rates over 95% in at least 10 years follow-up.^2^ ^-^ ^5^ However, a small portion of TKA show failure over time, needing revison.^6^ These failures are characterized by pain, functional disability of the knee and/or radiographic evidence of release of one or more components.^7^

With the increased life expectancy and a larger number of primary surgeries, consequently, a larger quantity of revision surgeries must be performed. Kurtz et al.^8^ estimate that TKA revisions in the USA will increase 600% by 2030. According to the National Center of Health Statistics 381,000 TKA were carried out in 2003 in the USA, and according to the American Association of Orthopedic Surgeons (AAOS), and 475,000 shall be performed by 2030. In 2003 35,000 revision TKA surgeries were performed, i.e. 9% of all arthroplasties were revision surgeries. For 2030 it is estimated that 43,500 revision surgeries will take place in the USA.^9^

On failure of a primary TKA, revision surgery is performed, usually, with intramedullary nails among the components to increase the stability of the femoral and tibial implants, besides metal wedges for filling of bone defects. In major failures, structured allograft or trabecular metal wedges can also be used.^10^

In cases of large bone defects with loss of ligament insertions on the femur or tibia, significant ligament insufficiencies or gross imbalance between the extension and flexion spaces, conventional revision implants do not promote stability in appropriate varus-valgus, making the revision arthroplasty unstable.^11^ As a solution to this problem, hinge type arthroplasty systems have been developed with excellent results in recent studies.^12^ ^-^ ^15^

**Figure 1:**
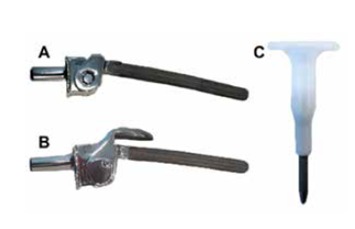
Hinge implant associated with rotating tibial basis (IMPOL, São Bernardo, SP, Brazil). A) Hinge; B) Hinge with shed; C) Tibial base.

Complex systems, however, are not always available in all countries due to their high cost and difficulty to import from foreign countries, therefore, complex revision cases should be solved with locally available implants in order to avoid non-conventional tumor prosthesis.^16^ The hinge implant associated with a rotating tibial basis (Figures 1 and ^2^) is proposed as a possible cost effective solution for knee revision arthroplasty with large bone loss or gross ligamentous instability.

**Figure 2:**
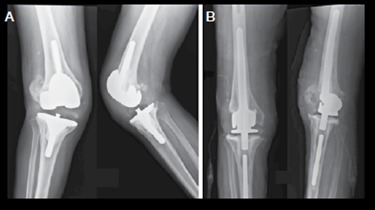
A) Seventy two year old patient revision prosthesis held in 2012 at another service, with late postoperative infection associated with ligament failure; B) Patient was subjected to implant removal and spacer placement and subsequent revision implant with hinge associated to rotating tibial base. After 1 year postoperatively patient presented range of motion 0°-110°.

The aim of this study was to evaluate the results, with at least two years follow-up, of TKA revisions performed with hinge implant associated to a rotating tibial basis in high complexity cases.

## MATERIALS AND METHODS

Patients who underwent TKA revision using hinge associated to rotating tibial basis (IMPOL, São Bernardo, SP, Brazil), between 2003 and 2013 were retrospectively evaluated.

The study was approved by the Scientific Committee of the Department of Orthopedics and Traumatology of the institutions (protocol number 1130). Patients were called for clinical evaluation update and oriented on the study. All participants signed a Free and Informed Consent Term.

Patients who met the following criteria were included: those undergoing knee arthroplasty review with hinge implant associated with tibial rotational basis, any age and gender and with at least two years of follow-up. Exclusion criteria were: lack of data in medical records that made it impossible to fill the desired information, not signing or not understanding the Free and Informed Consent.

Information collected from medical records included age, gender, comorbidities, reason to indication and date of surgery, laterality, knee range of motion in the postoperative follow-up and complications in the postoperative period.

Information collected from patients included clinical and functional evaluation by applying the Portuguese version (KSS-pt) of the Knee Society Score^17^ and the Portuguese version of Knee Injury and Osteoarthritis Outcome Score (KOOS-pt).^18^

## RESULTS

During the period from 2003 to 2013, 14 patients underwent revision knee arthroplasty with hinge implant associated with rotating tibial basis three of which were excluded due to missing data in the medical records and patients' unavailability to perform clinical evaluation update. Among the eleven patients included in the study, six were male and five female, with mean age at time of surgery of 70 years old (range 53 - 83 years old). (Table 1)

**Table 1:** Epidemiological data.

Patient	Age at surgery (years old)	Current age (years old)	Gender	Comorbidities	Side
1	65	77	F	SAH	R
2	59	66	M	RA, glaucoma, cataract	R
3	77	84	F	None	L
4	83	90	F	AF	R
5	77	82	M	SAH, COPD, RA	R
6	82	87	F	SAH, cardiomyopathy	L
7	74	77	F	SAH, cardiomyopathy	L
8	66	68	M	SAH, RA	R
9	53	55	M	None	R
10	71	73	M	SAH, Parkinson, DLP	R
11	61	63	M	none	R
Mean	70	75
SD	9,34	10,38

SAH: Systemic arterial hypertension; DLP: Dyslipidemia; RA: Rheumatoid arthritis; AF: Atrial fibrillation; COPD: Chronic obstructive pulmonary disease; SD: Standard deviation.

In all cases included in the study, indication of the aforementioned implant was due to large bone defects with loss of ligament insertions on the femur or tibia after removal of previous primary or revision knee arthroplasty, considered type III, according the classification of bone defects in the knee developed by the Anderson Orthopedic Research Institute (AORI).^19^ The cause of arthroplasty removal was septic loosening in four cases (36%), late infection in five cases (45%), instability in one case (9%), and one case (9%) of a periprosthetic fracture in a patient who already had septic loosening of TKA and was awaiting revision surgery. Of the four cases of loosening, three were already revision implants. (Table 2)

**Table 2:** Indications and complications.

Patient	Indication	Complication	Postoperative period	Treatment / Evolution
1	Instability	None	---	---
2	Late postoperative infection	Postoperative infection	1 month - 1 year	Implant removal; transfemoral amputation
3	Late postoperative infection	Erysipelas	< 1 month	Antibiotics
Septic loosening	Postoperative infection	> 1 year	Surgical cleaning
4	Late postoperative infection	Breaking of the tibial component	> 1 year	Revision of the tibial component
5	Septic loosening	Postoperative infection	< 1 month	Surgical cleaning
6	Septic loosening + periprosthetic fracture	None	---	---
7	Septic loosening	None	---	---
8	Late postoperative infection	Postoperative infection	---	Surgical cleaning; death
9	Septic loosening	Breaking of the polyethylene	1 month - 1 year	Implant revision
10	Late postoperative infection	Intra-operative culture positive	< 1 month	Antibiotics
11	Instability	Postoperative infection	< 1 month	Surgical cleaning and rotation of gastrocnemius flap

**Figure 3:**
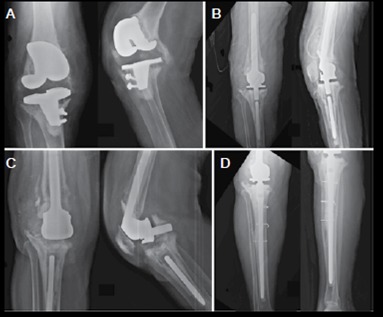
A) Fifty five year old patient with septic loosening of TKA performed at another service; B) Patient underwent implant removal with spacer placement and further review with hinge associated with rotating tibial basis; C) Patient suffered falling to the ground, breaking the polyethylene tibial base; D) Patient submitted to revision of the tibial component with a long shaft.

Three patients presented with no complications until the last evaluation. One patient had a positive intraoperative culture and was treated with antibiotics for six months. One patient presented with erysipelas in the first postoperative month and was also treated with antibiotics; this patient had an acute late postoperative infection after two years, being treated with a single surgical cleaning and evolving well. Two patients had acute postoperative infection in less than one month after surgery and were treated with surgical cleaning; one of them required gastrocnemius flap rotation for covering after debridement; the two had good evolution without remission of infection. One patient developed acute postoperative infection that required implant removal and evolved into transfemoral amputation of the affected limb. One patient, also with acute postoperative infection, underwent several surgical cleanings, but evolved with septic shock and died. Two patients had complications related to the implant integrity following trauma; one suffered a fall one year later and presented breach of the polyethylene rotatory tibial basis, requiring implant revision(Figure 3); another patient also suffered a fall three years later, progressing to breakage of the tibial component stem and needed revision surgery. (Table 2)

The present surgery follow-up period ranged from two to twelve years. KSS-pt varied from 67 to 95 points. KOOS-pt ranged from 27 to 35 regarding pain, 50-60 for activities of daily living, 0-2 for sport and leisure, 3-12 for quality of life, and 21-28 for other symptoms. (Table 3)

**Table 3:** Clinical-functional assessment.

Patient	ROM at the first year		Years PO	KSS-PT	KOOS-PT	
	1 month	6 months	1 year	Pain	DLA	SL	QL	O
1	5-90^o^	5-90^o^	5-90^o^	12	85	34	57	0	10	26
2	---	---	---	7	Amputee					
3	10-90^o^	0-85^o^	0-85^o^	7	67	27	50	0	3	23
4	0-90^o^	0-90^o^	0-90^o^	7	85	33	55	0	4	25
5	0-55^o^	0-100^o^	0-100^o^	5	95	35	52	0	12	28
6	0-15^o^	0-90^o^	0-90^o^	5	85	30	54	0	6	23
7	0-90^o^	0-85^o^	0-85^o^	3	88	33	60	1	11	27
8	---	---	---	---	Death					
9	0-90^o^	0-90^o^	0-90^o^	2	83	32	59	1	5	21
10	0-45^o^	0-90^o^	0-110^o^	2	94	36	38	2	8	25
11	5^o^ -95^o^	5-90^o^	5-90^o^	2	87	34	58	2	8	27

ROM: Range of movement; PO: Postoperative; DLA: Daily life activities; SL: Sports and leisure; QL: Quality of life; O: Other symptoms.

## DISCUSSION

The amount of TKA revision surgeries is increasing due to increased life expectancy and the higher number of primary surgeries performed.^8^ Hinge type contrite implants with rotating components are a good option for TKA revision where there is significative bone loss and ligament instability.^12^ ^-^ ^15^ ^,^ ^20^ ^,^ ^21^ Sanguineti et al.,^12^ with 20 cases of TKA revision surgeries using the Endo-Model rotating-hinge prosthesis (Waldemar Link GmbH and Co., Hamburg, Germany), Neumann et al.^22^, with 24 cases using the NexGen Rotating Hinge Knee (Zimmer Biomet Inc., Warsaw, IN, USA) and Bistolfi et al.^14^ with 50 cases also using the Endo-Model prosthesis, are some of the largest series showing the follow-up of patients submitted to TKA revision with this type of implant. These implants, however, although also of the hinge type, have a quite different design than the implants used in this study. Similarly to our study, the indications for revision TKA in these studies ranged from postoperative infection, implant loosening and periprosthetic fracture.

Sanguineti et al.^12^ reported 20 cases with five years follow-up, with only two complications in the period, a dislocation of the implant by trauma occurred in rehabilitation and a deep infection.

Neumann et al.^22^ reported 20 cases with a minimum follow-up of 36 months. There were two cases in which there were radiolucent lines around the tibial component and a case of patellofemoral subluxation, which was treated with revision of the patellar component, lateral release and advance of the vastus medialis.

The work of Bistolfi et al.^14^ was the series with more cases and longer follow-up: 50 cases, of which 34 had minimum follow-up of 150 months. As early complications the authors observed four cases of wound dehiscence, two cases of hematoma and two cases of infection, the latter treated with debridement, polyethylene exchange and antibiotics. As late complications, nine cases of polyethylene breaking were observed. In three cases, revision of the implant was necessary due to generated instability. In both cases, the polyethylene wear and breakage occurred due to dislocation and in one case, due to septic loosening. In the other three cases the damage to the polyethylene was asymptomatic and the conduct was conservative. There were two cases of poor functional outcome - a case of injury to the extensor mechanism and one case of septic loosening - treated conservatively due to poor clinical condition of the patients.^14^

The complication rate in our study was high. The most frequent complication diagnosed was postoperative infection, which can be attributed to the following factors: approximately 45% of patients had revision surgery indicated by septic loosening; the profile of the enrolled patients (advanced age, ill health and previous comorbidities, lower social and instruction level). Still, most patients who developed infection were treated successfully with surgical cleaning and proper use of antibiotics. Because ours is a reference service for high complexity cases, most patients with poor functional outcomes or arthroplasty failures ended up being referred to our hospital, therefore, most of the cases that were reviewed due to infection presented the primary condition in a different service.

In two cases there was breakage of the implant component. We believe that, in these cases, failure of the implant was due to fatigue caused by increased stress due to ligament absence, in which the stress transfer in the bone-cement-implant interface is high. In recent studies, which used more developed implant models, this was not a frequent complication.^13^ ^,^ ^14^ ^,^ ^22^ Because of this potential complication, we advise all patients to use, postoperative, continuous and permanent support to walk (e.g. a cane), in order to provide maximum protection for the operated limb and the implant.

Due to the shape of the implant, which has the anterior part of the femoral component squared, we frequently observed dislocation of the patella upon knee flexion intraoperatively. A lateral wide release was necessary in all cases to fix the patellar tracking. Nevertheless, none of the patients assessed complained of pain in the anterior knee and all managed at least 85° flexion after one year postoperatively, showing that the potential increase of patellofemoral pressure did not lead to loss of range of motion.

The mean age in our study was high (70 years), similarly to the literature. Older patients showed generally good results regarding the expectations of performing activities of daily living, quality of life, and little complaint about symptoms such as pain, instability or stiffness. The presented functional outcomes can be considered acceptable for patients with low functional demands, but for patients with moderate to high functional demands, the implant may not match the treatment expectations. Nevertheless, in our series, five patients were younger than 70 years old at time of surgery and three of them who could be evaluated had KSS greater than 80.

The limitations of this study were its retrospective nature and low number of patients. Anyway, due to shortage of implants for the treatment of instabilities and coarse bone loss in our midst, as well as the relatively small number of complications, it is important to know the functional outcome of patients using simple design constricted articulated hinge implants associated to rotating tibial basis.

## CONCLUSION

In the absence of newer design implants, the constricted hinge implant associated to rotating tibial base is a viable option for revision TKA where there is significant bone loss and ligamentous instability, especially in patients with low functional demand. Despite the observed complications rate, the functional outcome was considered acceptable for most patients.
